# Comparing MEG and EEG measurement set-ups for a brain–computer interface based on selective auditory attention

**DOI:** 10.1371/journal.pone.0319328

**Published:** 2025-04-10

**Authors:** Dovilė Kurmanavičiūtė, Hanna Kataja, Lauri Parkkonen

**Affiliations:** 1 Department of Neuroscience and Biomedical Engineering, Aalto University, Finland; 2 Aalto NeuroImaging, Aalto University, Finland; Indian Institute of Technology Guwahati, INDIA

## Abstract

Auditory attention modulates auditory evoked responses to target vs. non-target sounds in electro- and magnetoencephalographic (EEG/MEG) recordings. Employing whole-scalp MEG recordings and oﬄine classification algorithms has been shown to enable high accuracy in tracking the target of auditory attention. Here, we investigated the decrease in accuracy when moving from the whole-scalp MEG to lower channel count EEG recordings and when training the classifier only from the initial or middle part of the recording instead of extracting training trials throughout the recording. To this end, we recorded simultaneous MEG (306 channels) and EEG (64 channels) in 18 healthy volunteers while presented with concurrent streams of spoken “Yes”/“No” words and instructed to attend to one of them. We then trained support vector machine classifiers to predict the target of attention from unaveraged trials of MEG/EEG. Classifiers were trained on 204 MEG gradiometers or on EEG with 64, 30, nine or three channels with trials extracted randomly across or only from the beginning of the recording. The highest classification accuracy, 73.2% on average across the participants for one-second trials, was obtained with MEG when the training trials were randomly extracted throughout the recording. With EEG, the accuracy was 69%, 69%, 66%, and 61% when using 64, 30, nine, and three channels, respectively. When training the classifiers with the same amount of data but extracted only from the beginning of the recording, the accuracy dropped by 11%-units on average, causing the result from the three-channel EEG to fall below the chance level. The combination of five consecutive trials partially compensated for this drop such that it was one to 5%-units. Although moving from whole-scalp MEG to EEG reduces classification accuracy, usable auditory-attention-based brain-computer interfaces can be implemented with a small set of optimally placed EEG channels.

## Introduction

Selective auditory attention is a cognitive process that allows individuals to focus on a specific sound source while filtering out competing background noises or irrelevant auditory stimuli. To study this process, a dichotic listening task [1] is often employed; the subject is presented with two concurrent auditory streams – one for each ear – and is instructed to follow one of them. Attention to a specific, behaviorally relevant stream or speaker is considered to enhance its representation in the auditory system [2–4]. The corresponding changes in brain responses induced by such attentional selection can be recorded non-invasively by magneto- or electroencephalography (MEG / EEG) [2,5–8], whose millisecond-level temporal resolution enables tracking auditory processing as it unfolds.

Such attention-dependent modulation can be exploited in a brain–computer interface (BCI) based on EEG or MEG measurements [9–12]. Although MEG is usually not the primary choice for a BCI due to its cost and non-portability, it may provide more information than EEG on the relevant brain processes and serve as an efficient development platform for a BCI that will eventually be deployed as EEG-based [13,14].

Our previous MEG study [15] using a modified dichotic listening task, where participants were presented with two simultaneous streams of spoken-word stimuli, demonstrated that the target of selective auditory attention can be detected with high accuracy from just 1 s of MEG data. However, in this study, as in most MEG/EEG classification studies, the classifier was trained with data randomly sampled throughout the recording. Although such a training can be considered optimal, it cannot be applied to a real-time BCI as there the training can happen only with data from the beginning of the measurement session.

Previous studies using auditory EEG-BCI have shown promising results in target detection in healthy participants [16–21] and patients [22]. The precision of such a BCI naturally depends on the ability of the classifier to extract relevant brain activity [23,24]. Brain activity is influenced by the experimental design and the measurement setup [25]: the number of training trials and their distribution throughout the session, the duration of each trial, as well as the number, type, and location of the measuring channels. All of these design properties are known to affect the classification accuracy, but it is not known to what extent.

To explore the effect of these parameters, we recorded simultaneous MEG-EEG while the participants performed a selective attention task. We investigated how much the classification accuracy decreases when the classifiers are trained only on trials from the beginning of the recording (as in the calibration phase of an actual BCI system) instead of using the same number of trials randomly extracted from the entire recordings (as in typical oﬄine classification). Furthermore, we tested whether training data extracted from later parts instead of the beginning of the measurement improves classification accuracy. In addition, we tested how the classification accuracy depends on the type (MEG vs. EEG) and the number of channels and on the number of trials used to train the classifier.

## Materials and methods

### Participants

Eighteen healthy adult volunteers (8 females; 10 males; mean age 28 . 8  ±  3 . 8 years, range 23–38 years) participated in the study from September 26 to October 31, 2017. According to the Edinburgh Handedness Inventory [26], one participant was left-handed, three were ambidextrous, and the rest were right-handed. Participants did not report hearing problems or a history of psychiatric disorders. The study (Statement 2017_15_Real-time detection and decoding of responses) was approved by the Aalto University Research Ethics Committee. The research was carried out according to the Declaration of Helsinki guidelines. Written informed consent was obtained from each study participant using a paper-based consent form prior to measurements. One participant was excluded from MEG/EEG data analysis due to misunderstanding the task, one was excluded from MEG data analysis due to poor data quality, and six more were excluded from EEG data analysis due to extensive artifacts in their data, leaving 16 participants for MEG and 11 participants for EEG. When comparing MEG and EEG, only data and results from the same participants were contrasted.

### Stimuli and experimental protocol

**Fig 1:**
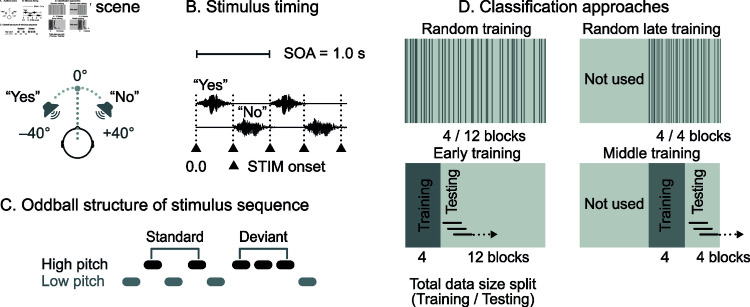
Experimental design and conditions. (**A**) Spoken-word stimuli recorded with a dummy head were presented to the participant. In the acoustic scene, the two speakers appeared at  ± 40 degrees of the participant’s head mid-line. (**B**) The stimulus onset asynchrony (SOA) was 1 s, leaving about 400-ms silent interval between consecutive stimuli. The black triangles indicate stimulus onsets. (**C**) The high- and low-pitch version of the words alternated (standard) but occasionally two same-pitch words were presented consecutively (deviant). (**D**) Classifiers were trained with trials extracted randomly across the entire recording (random training), or with trials only from the beginning of the recording (early training), or with trials extracted randomly from the latter half of the measurement (random late training), or with trials from the middle of recording (middle training).

The stimuli were adapted from our previous study [15]. The stimuli created an acoustically realistic scene with two speakers standing in fixed positions in front of the participant at –40 (left) and +40 degrees (right) from the mid-line, continuously uttering the words “Yes” (a female speaker) and “No” (a male speaker) in a sequence where high- and low-pitch versions of the stimulus word alternated. Both words were presented once per second, and the streams were interleaved such that the words had minimal overlap; see Fig 1. The word sequences contained occasional deviants (violations of the regular alternation) that comprised three consecutive high-pitch versions of the word and occurred with a 5% probability in each stream; see Fig 1C.

PsychoPy (version 1.79.01) [27,28] Python package was used to control the experiment and present the auditory stimuli and visual instructions. PsychoPy was run on Ubuntu Linux (version 14.04). Auditory stimuli were delivered by a professional audio card (E-MU 1616m PCIe; E-MU Systems, Scotts Valley, CA, USA), an audio power amplifier (LTO MACRO 830; Sekaku Electron Industry Co., Ltd, Taichung, Taiwan), and custom-built stereo loudspeaker units located outside of the shielded room. Two plastic tubes conveyed the stimuli to both ears separately. The sound pressure was adjusted to a comfortable level for each participant.

#### Experimental structure and the task

The experiment consisted of 16 blocks and, in total, with the breaks between the blocks, lasted 45 minutes.

The task of the participant was to focus on the spoken-word stream indicated in the visually presented instruction prior to each block and to minimize eye movements by maintaining gaze on the fixation cross displayed on the screen, which was 1.4 m from the eyes of the participant. If the cue “LEFT-YES” was displayed, the participants had to attend the “Yes” stream presented by the virtual speaker on their left side and, conversely, for the cue “RIGHT-NO”. Before measurements, participants were given two “practice blocks” to learn to focus on “Yes” or “No” streams. Unlike in our previous experiment with the same stimuli, here participants were not requested to count and report the deviants. The experiment always started with the block “LEFT-YES”, followed by block “RIGHT-NO”. The subsequent blocks were presented in random order for each participant.

### MEG/EEG data acquisition

MEG/EEG measurements were performed with a whole-scalp 306-channel Elekta–Neuromag VectorView MEG system (MEGIN Oy, Helsinki, Finland) at the MEG Core of Aalto Neuroimaging, Aalto University. All participants were measured in the seated position. In addition to MEG, simultaneous EEG was recorded with a 64-electrode Waveguard™ EEG cap (Advanced NeuroTechnology, Enschede, The Netherlands).

Anatomical landmarks (nasion, left, and right preauricular points), head position indicator coils, and additional scalp surface points (around 100) were digitized using an Isotrak 3D digitizer (Polhemus Navigational Sciences, Colchester, VT, USA). A bipolar electro-oculogram (EOG) with electrodes positioned around the right eye (laterally and below) was recorded. The MEG / EEG data were filtered to 0.03–330 Hz and sampled at 1 kHz during recording.

### Analysis

The analysis pipeline was implemented using the MNE-Python (version 0.17) [29] and scikit-learn (version 0.18) [30] software packages.

#### Pre-processing.

External magnetic interference in MEG signals was suppressed using signal-space separation [31] as implemented in MaxFilter software (version 2.2.10; MEGIN Oy, Helsinki, Finland). No continuous head movement correction was performed because the MEG data were recorded without continuous head position tracking. Only the 204 planar gradiometer channels were retained for further analysis.

The EEG data were re-referenced using the reference electrode standardization technique (REST) after removing bad channels (see Ref. [32] for more details). Bad channels were not interpolated because averaging neighboring activity to recover the bad channel(s) would not add additional information for the classifiers. The number of bad channels varied from 2 to 11 (only one participant), on average 4.3 channels, which left on average 60 good channels for EEG analysis; however, in the text EEG will always be referred to as 64-channel EEG.

The unaveraged MEG-EEG data were down-sampled to 125 Hz and filtered to 0.1–45 Hz. The ocular and cardiac artifacts were suppressed by removing the independent components (3–10 per participant, on average 6) that correlated the most with the EOG or ECG signals, respectively. One-second epochs (–0.2, 0.8 s) were extracted from the MEG/EEG data around every stimulus, and the first 0.2 s were used as the baseline. Individual epochs were discarded if their signal amplitude exceeded 5 pT for magnetometers, 4pT/cm for gradiometers, 80*μ*V for EEG or 150*μ*V for EOG, which can be considered to result from artifacts. Epochs corresponding to deviants were excluded from further analysis.

Originally, one measurement block comprised 480 trials. Due to rejecting the deviants and artifacts, on average 356 trials (range 268–422 trials) were accepted per block.

#### Classification.

Before passing the MEG/EEG signals to the classifier, their mean was removed and they were scaled to unit variance. This scaling was applied to the entire feature at once, thus preserving the spatial and temporal distribution of the signals. All time samples of the included MEG (204 planar gradiometers) or EEG (3–64) channels were concatenated to form the feature vector for classification. Thus, the classifier had both temporal and spatial information about the brain responses.

Two linear-kernel support vector machines (SVM) [33] were applied to the down-sampled epochs for the classification task. One SVM was used to classify attended vs. unattended stimuli in the “Yes” spoken-word stream and similarly another one for the “No” stream. The SVM regularization parameter C was set to 1.0.

We trained the SVM classifiers in two different ways. First, similarly to the calibration phase of an actual BCI, we trained the classifiers with the data trials extracted only from the beginning of the measurement (corresponding to the first four data blocks B1–B4), later referred to as *early training*; see Fig 1D. Second, we trained the classifiers by randomly picking the training data trials throughout the measurement, later referred to as *random training*; see Fig 1D. Consequently, we trained classifiers with trials of the middle four (B9–B12) blocks, later referred to as *middle training*. Finally, classifiers trained with trials randomly selected from the latter half of the data (B9–B16) are referred to as *random late training*; see Fig 1D. In all four training modes, 25% of the data, which is equal to four data blocks, were used for training.

The early-training classifier used test data from blocks B5–B16 and middle training from B13–B16 in three different ways. In the first way, the classification results for five consecutive trials were combined to yield a confidence value ranging from 0 (full confidence for the attended word stimuli) to 1 (full confidence for the unattended stream). The value of 0.5 indicated that the classifier did not have information on the direction of auditory attention. This combination was done as a moving average that spun five trials (5 s of data) at a time.These confidence values were then converted to binary values based on which the accuracy of the classifier was calculated. The final accuracy was obtained by taking the mean accuracy of the classifiers for the “Yes” and “No” streams. The second way was similar to the first one, except that only two consecutive trials were combined. Finally, the third way was to employ only single trials.

**Fig 2:**
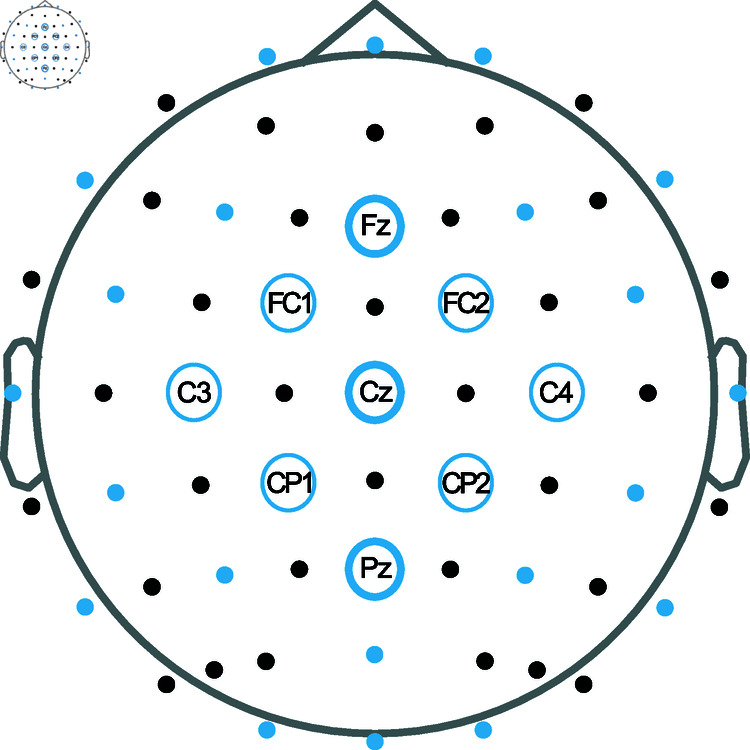
Layout of the EEG channel selections in the measurement. The 64-channel EEG set (all dots), and the subsets of 30 (blue dots and all circles), nine (all blue circles) and three (thick blue circles) channels.

The effect of varying the number of EEG channels used for the classification was investigated by restricting the original 64-channel EEG to 30 channels (electrode locations according to the international 10–20 system), to nine channels (*Fz, FC1, FC2, Cz , C3, C4, CP1, CP2, Pz*) and to three channels (*Fz, Cz, Pz*). The nine and three channel selections were determined based on a study by Sellers and colleagues [34]. The different sets of EEG channels used in a classification are shown in Fig 2.

The dependence of the classification accuracy on the amount of training data was tested by varying the number of training trials given to the two SVM classifiers. To this end, the training data set (750 trials) and the testing data set (250 trials) were obtained on randomly shuﬄed data. The classifier training started with 10 trials and gradually increased, with a step of 50 trials, to the maximum number of training data trials (750 trials). The classifiers, trained on each training set, were tested with 250 test trials. The procedure was repeated for 20 times for each training set size. The average classification accuracy and the standard error of mean were calculated for each training set size for those 20 repetitions and across all subjects (*N*=11). Finally, the average accuracy was obtained for the 204-channel MEG and 64-, 30-, nine-, and three-channel EEG selections.

## Results

**Fig 3:**
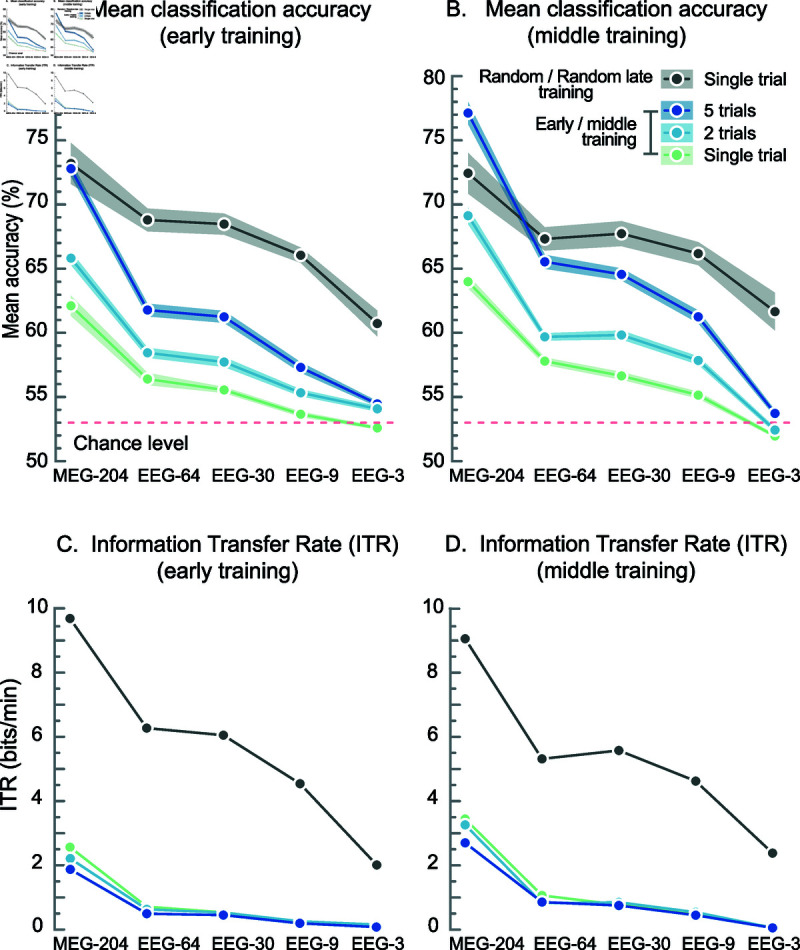
Classification accuracy and information transfer rate (ITR) as a function of the measurement set-up and the number of combined trials. (**A**) Classification accuracy when classifiers were trained with 25% of the data extracted randomly across the entire recording (grey) or with only the first quarter of the data (blue, green). (**B**) Classification accuracy when classifiers were trained with the middle blocks B9–B12 and tested with last four blocks B13–B16. The dots in panels A and B represent the mean accuracy across the 11 participants, and the shading the standard error of mean. (**C**) Information transfer rate as bits/min with training as in panel A. (**D**) Same as in Panel C but training as in Panel B. The dots in Panels C and D represent the mean ITR across the 11 participants, and the shading the standard error of mean.

MEG yielded the highest classification accuracy, reaching 73.2% for single (1.0-s) trials when training with randomly extracted trials; see Fig 3. Under the same conditions, the 64-channel EEG provided a slightly lower accuracy of 68.8%, which decreased only little when the number of channels was reduced to 30 (68.5%) and nine (66.1%) but dropped substantially with only three channels (60.7%). With the random late training, the accuracy of the classification reached 72.5% for MEG, 67.3% for 64-channel EEG, 67.8% for 30, 66.2% for nine, and 61.7% for three-channel EEG; see Fig 3B.

Classifiers trained with trials extracted randomly across the recording (as is customary in oﬄine classification studies) produced a systematically higher accuracy than those trained with the same number of trials only from the beginning of the recording (as must be done in an online BCI application); see Fig 3A and 3B. This difference was on average 11 and 10%-units (range 8–13 and 8–11%-units), respectively, for the early and middle training across the different number of channels used.

Combining the classification results of consecutive trials improved the overall classification accuracy as expected. Here, using two trials increased the accuracy by around 2%-units on average across measurement setups and using five trials by around 5%-units. The classification accuracy improved further if classifiers were trained on data from the middle of the recording and trials were combined; the accuracy for five combined trials rose by about 4%-units compared to early training.

The information transfer rate (ITR) estimated from the accuracy shown in Fig 3A was by far the highest for the random-training classification. All ITR estimates increased systematically with more measuring channels, but the greatest increase was observed between 64-channel EEG and 204-channel MEG; see Fig 3C. In the middle-training mode, the ITR values increased with the increased accuracy compared to the early-training mode. Only the ITR of the random late training was lower than that of the full-data random training; see Fig 3C and 3D.

**Fig 4:**
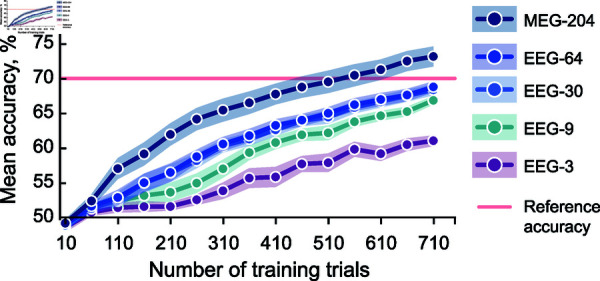
Classification accuracy as a function of the amount of training data. The dots in the learning curves represent the mean classification accuracy across the 11 participants. The shading indicates the standard error of mean calculated over 20 iterations to each training set size.

To find the required number of training trials for a given decoding accuracy, we trained a classifier with a variable number of data trials. All variations in training data size were tested on other 250 data trials. The accuracy obtained for each size of the training data is shown in Fig 4. The classification results show that to achieve 70% accuracy on the 204-channel MEG at the group level requires about 560 training trials (corresponding to about 9.3-min in our paradigm). However, the 70-% reference accuracy was not achieved on the EEG data within the maximum training data size.

## Discussion

In this study, we investigated how the performance of an auditory-attention-based BCI depends on the measurement modality (MEG vs EEG), the number of measurement channels (EEG), the number of trials combined for a classification result, the number of training trials and whether they are extracted randomly across or only from the beginning of the recording as must be done in an actual BCI.

MEG yielded a significantly better classification accuracy than the EEG set-ups we included in this study. Since we utilized 204 MEG channels and at maximum only 64 EEG channels, this comparison does not directly tell about the relative performance of MEG vs. EEG. However, since increasing the EEG channel count from 30 to 64 led only to a small increase in classification accuracy, we would anticipate that increasing the EEG channel count further to 128 or even higher would not make EEG outperform MEG. Hence, the observed advantage of MEG is likely related to higher signal-to-noise ratio and better spatial separability of cortical sources compared to EEG. However, these advantages of MEG over EEG come with the cost and non-portability of the system. Because of this reason, MEG is not currently well suited for BCI outside of the laboratory environment.

Clinical EEG measurements are sometimes performed with just a few EEG channels. According to our results, reducing the number of EEG channels below 30 significantly affects the classification accuracy and therefore a reduced clinical EEG set-up may not be sufficient for a BCI based on selective auditory attention. Indeed, our results with a nine-channel EEG did not indicate sufficient performance for a practical BCI even though the locations of these channels were optimized for this task based on a previous study by Sellers et al. [35]. However, surprisingly, oﬄine classification (training trials extracted randomly throughout the recording) yielded acceptable performance with just those nine channels. The classification of the attention direction using the three-channel EEG did not exceed the chance level even when combining trials.

The classification accuracy obtained with the 64- and 30-channel EEG was rather similar, indicating that a 30-channel system—even without task-optimized electrode locations—is already able to sample most of the information available in the scalp EEG about the direction of auditory attention. These results support a previous study that examined the optimal number of EEG channels for comparable classification tasks; they have shown that the marginal utility of additional channels drops above about 22 channels [24]. Another interesting result in that study was that a generic EEG channel layout does not perform well with all subjects. Instead, individual EEG channel layouts should be favored for optimal performance. Such optimization may be time consuming but is likely worthwhile if the BCI is to be used for a long time.

In addition to optimal channel selections, classifier training approaches have a clear effect on classification accuracy. For example, cross-validation-type training and testing gave a substantially higher accuracy than training with the same number of trials exclusively from the beginning of the recording. This difference could be due to the participants changing their strategy of attending one stimulus stream in the course of the experiment. However, it could also arise from changes in background brain activity, physiological artifacts, and ambient interference, which would make training the classifier based only on the beginning of the measurement sub-optimal. A likely neurophysiological change across the recording is the increase in the alpha- and beta-band spontaneous brain activity as the participant becomes more relaxed or even drowsy as the measurement progresses and the initial excitement is over. In our study, using the middle data blocks to train the classifiers improved classification accuracy with respect to training with the early blocks, providing further evidence of high physiological variability at the beginning compared to the later parts of the measurement.

By varying the number of training trials, we showed that to achieve 70% accuracy with 204-channel MEG would require 560 trials, on average 152 trials less than our full training data set (on average 712 trials). With 64 and 30 EEG channels, using all the 710 training trials yielded an accuracy close to 70%. In contrast, classification with nine channels EEG required more than 710 trials to arrive at that accuracy, and with just three EEG channels such an accuracy seems unreachable.

However, the loss of classification accuracy due to fewer measurement channels or shorter training time can be partially compensated for by combining classification results of consecutive trials at the expense of the information transfer rate (ITR) such a BCI can provide. In general, combining individual trials increases the classification accuracy only if there is no systematic classification error. Our results show no considerable bias in the classification results of individual trials, since we observed a clear and monotonic increase in accuracy when adding more trials to the combination.

The ITR the using early training classification was low for EEG channel selections and four to five times higher for MEG. Adding more trials increased ITR for MEG but not for EEG. However, the best ITR was for the classification accuracy obtained using single trials with random training. When middle training classifiers were used, the ITR almost doubled, except for random late training. Thus, the take-off of these results is that a priori knowledge on how data will behave secures the best accuracy and ITR, whereas if such information is unavailable as in real-time BCIs, the longer training and habituation could be beneficial to obtain better accuracy and ITR. Regarding multiple trial influence to classification accuracy, one possibly could optimize, especially when developing real-time BCI. Therefore, to optimize the trade-off between ITR and classification accuracy, da Cruz and colleagues [36] proposed an adaptive decision time window, which improved ITR by 19% in their BCI based on steady-state visual evoked potentials. In our experimental design, an adaptive time window could be realized by accumulating classification results from as many trials as needed to reach a certain confidence level, determined based on the class probabilities given by the SVM algorithm.

In this study, we did not aim for a BCI that generalizes across participants but instead trained the BCI individually for each participant. This individual training has also been shown to yield higher performance in certain patient populations [37]. However, the ability of the BCI to generalize across participants could still be a useful feature [38,39] since the individual tuning of a BCI takes time. However, with successful calibration, such a BCI typically provides a substantially higher classification accuracy than a BCI generalizing across subjects.

In this study, we did not focus on nor evaluate factors such as motivation, mental fatigue, frustration, anxiety [40–43], or the mental strategy, which the participants used to focus their attention. These factors may have an effect on the classification accuracy [43]. Moreover, we relied on our participants understanding the task and performing it as instructed.

The current development of new types of MEG sensors, particularly optically pumped magnetometers (OPM), may enable wider adoption of MEG in BCI applications, although magnetic shielding is still required with these novel sensors [44,45]. As demonstrated by our results on MEG data, such OPM-MEG systems may provide substantially higher performance than any EEG set-up.

## Conclusion

In conclusion, we have demonstrated how training time and moving from a whole-scalp MEG to EEG with different channel counts affect classification accuracy in an auditory-attention-based BCI. Our results provide guidelines on how to reach the desired classification accuracy given the measurement set-up.
